# Challenges and innovation: Disease modeling using human-induced pluripotent stem cell-derived cardiomyocytes

**DOI:** 10.3389/fcvm.2022.966094

**Published:** 2022-08-12

**Authors:** Louise Reilly, Saba Munawar, Jianhua Zhang, Wendy C. Crone, Lee L. Eckhardt

**Affiliations:** ^1^Cellular and Molecular Arrhythmia Research Program, Division of Cardiovascular Medicine, Department of Medicine, University of Wisconsin-Madison, Madison, WI, United States; ^2^Department of Engineering Physics, College of Engineering, University of Wisconsin-Madison, Madison, WI, United States

**Keywords:** stem cell, genomics, regeneration, arrhythmia modeling, ion channel, channelopathies, biotechnology, cardiac metabolism

## Abstract

Disease modeling using human-induced pluripotent stem cell-derived cardiomyocytes (hiPSC-CMs) has both challenges and promise. While patient-derived iPSC-CMs provide a unique opportunity for disease modeling with isogenic cells, the challenge is that these cells still demonstrate distinct properties which make it functionally less akin to adult cardiomyocytes. In response to this challenge, numerous innovations in differentiation and modification of hiPSC-CMs and culture techniques have been developed. Here, we provide a focused commentary on hiPSC-CMs for use in disease modeling, the progress made in generating electrically and metabolically mature hiPSC-CMs and enabling investigative platforms. The solutions are bringing us closer to the promise of modeling heart disease using human cells *in vitro*.

## Introduction

Before the discovery of human pluripotent stem cells (hPSCs), including human embryonic stem cells (hESCs) first isolated by Dr. James Thomson (UW–Madison) (1) and human-induced pluripotent stem cells (hiPSCs) created by both Dr. Shinya Yamanaka (Japan) and the Thomson group (2, 3), disease modeling had been limited to transgenic animal models and heterologous expression studies. These cells presented many advantages such as unlimited proliferation, the potential to differentiate to any cell types of the human body, and the generation of patient-specific isogenic cells for disease modeling. For the first time, we could generate human cardiac cells from healthy donors and patients following a skin biopsy or blood draw to reprogram the somatic cells to iPSCs which can be differentiated to cardiac cells (4). However, initial enthusiasm was tempered by the realization that the differentiated hPSC-cardiomyocytes (hPSC-CMs) did not quite resemble the adult cardiomyocytes physically, electrically, and functionally. To improve this powerful model system, extensive work has been done to promote hPSC-CMs maturation and differentiate to chamber-specific cardiomyocytes. In this focused commentary, we will discuss the benefits of this model and the challenges and creative technologies that have ushered in the next phases in utility for cardiac disease modeling.

## Human pluripotent stem cell-derived cardiomyocytes

Using hiPSCs has enabled the investigation of patient-specific conditions in a lab environment (4). Methods and protocols to differentiate hPSC-CMs have been significantly advanced in the past two decades, particularly in the past 10 years. From the beginning of the inductive co-culture and the embryonic body (EB) method, which mimic the formation of the three germ layers of ectoderm, mesoderm (from which CMs are derived), and endoderm, and spontaneous differentiation in undefined conditions, to the monolayer-based directed differentiation driven by growth factors or small molecules using defined media, the differentiation efficiency has been dramatically improved. By the EB spontaneous differentiation in undefined condition, only 5% hPSC-CMs could be obtained, but now to the monolayer-based, small molecule-directed differentiation of 95% hPSC-CMs can be generated in a cost-effective way (5–7). Differentiation of hPSC-CMs is guided by mammalian heart development from the generation of mesoderm, cardiac mesoderm, and heart field progenitors to differentiation of embryonic-like cardiomyocytes. Although with the significantly improved differentiation efficiency, the differentiation protocols we have developed and by other laboratories could generate still immature cardiomyocytes like in the fetal heart, and a mixture of ventricular, atrial, and nodal cells (4, 6, 8–11). However, great effort has been made to promote the maturation of hPSC-CMs (12–16) in recent years, which we will discuss in the later sections.

Different forms of heart disease target different regions and CM subtypes in the heart; e.g., long QT syndrome (LQTS) primarily targets on the left ventricle (LV), while Brugada syndrome (BrS) and arrhythmogenic right ventricular cardiomyopathy (ARVC) mainly affect right ventricle (RV), and atrial fibrillation impacts on atria. Therefore, the generation of chamber-specific hPSC-CM subtypes is needed to model specific heart disease and to develop novel therapies and precision medicine. The hPSC-CM differentiation protocols that have been used in many labs primarily generated majority of ventricular CMs (6, 8, 9). However, it has shown variations from lab to lab and from line to line, which is the challenge we face. Pacemaker cells shown could be differentiated using the EB protocol developed in Keller's lab (17). Lee et al. have also shown that human ventricular and atrial cardiomyocytes were derived from different mesoderm populations based on CD235a and RALDH2 expression in the early stage of hPSC differentiation, which could be further directed to differentiate to ventricular and atrial hPSC-CMs (18). However, how these mesodermal progenitors were specified to first heart field (FHF) and second heart field (SHF), and how the CD235a mesoderm progenitors contribute to FHF-derived left ventricle (LV) or SHF-derived right ventricle (RV) CMs or both were not clear (18). In one of our recent studies to differentiate hPSCs to cardiac fibroblasts, we found that FHF and SHF progenitors were differentiated in the early stage of either the biphasic Wnt signaling (GiWi) or the FGF signaling (GiFGF) protocols (19). More recently, Zhang et al. and Pezhouman et al. both created a hiPSC *TBX5*^*Clover*2^*/NKX2-5*^*TagRFP*^ and hES3*-TBX5*^*TdTomato*/*W*^*/NKX2-5*^*eGFP*/*W*^ double reporter line, respectively, for isolation of FHF- and SHF-derived hPSC-CMs (20, 21). However, delineating FHF and SHF lineages based on only the two transcription factors of *NKX2.5* and *TBX5* is not sufficient. Furthermore, different regions of SHF, anterior SHF (aSHF) and posterior (pSHF), contribute to different CM subtypes, in which aSHF gives rise to RV and outflow track (OFT) and pSHF contributes to atria (22–25).

To realize the full potential of using hPSC-CMs for disease modeling, drug screening, precision medicine, and cardiac regeneration largely rely on our ability to differentiate them to the ideal, specific, and closely akin to adult CMs. Since significant advancement and great progress have been achieved in the past 10 years, we have hope to make it even closer to the goal in the next 10 years.

## Modeling cardiac arrhythmia using HiPSC-CMs

Arguably, the most powerful modeling aspect of iPSCs is the capability of modeling human cardiac disease with human cardiac cells. This is a significant advance over animal models which have their unique physiology and cellular regulation. For example, resting heart rate in mice is 8–10 times faster than human, and ventricular repolarization is carried by potassium current (IK_to_ and IK_ur_) rather than delay rectifier channels I_kr_ or I_ks_ as in human (26, 27). The Ca^2+^ handling kinetics and myofilament proteins are also differentially expressed between human and mice. These features limit the capability of animal models to mimic human disease particularly when these currents are directly involved in the disease process or arrhythmogenesis.

Inherited arrhythmic syndromes are a broad disease category that implicate that abnormalities in cardiac ion channel α1-subunits, and proteins that associate with ion channels, are involved in contraction or are components of structural makeup (28). These diseases primarily involve ion channels, occur in otherwise healthy children and younger adults with structurally normal hearts, and present with a range of symptoms from palpitations to syncope to sudden death (29). The most common is LQTS with an incidence of about 1 of 2,000. LQTS cellular mechanism is due to ion channel dysfunction directly by mutations of ion channel α1-subunit, or ion channel associated proteins that alter channel function, or indirectly by mutations in protein structure that disrupts normal protein membrane trafficking. Other inherited cardiac arrhythmia syndromes known to involve ion channels include Brugada syndrome (30), cardiac conduction disease (31), catecholaminergic polymorphic ventricular tachycardia (CPVT) (32), calcium release disorder syndrome (CRDS) (33), and short QT syndrome (SQTS) (34). CPVT is linked to mutations in the ryanodine receptors (*RYR2*) and calsequestrin (*CASQ2*) genes that encode intracellular proteins involved in intracellular Ca^2+^ regulation (32). Many genes are implicated in several phenotypes including *SCN5a* that encodes for the α1-subunit for the cardiac sodium channel (Brugada syndrome, cardiac conduction disease, and LQTS), RYR2 (CPVT and CRDS), and hERG1 (LQTS and SQTS), which highlights the need for functional characterization combined with deep clinical phenotype assessment. Other non-ion channel-inherited arrhythmogenic syndromes can involve genes encoding structural and contractile elements in the heart, including arrhythmogenic cardiomyopathy (ACM) (includes ARVC) and laminopathy, and can first present with arrhythmia prior to structural remodeling likely related to the interplay of ion channel regulation with these proteins (35).

Several cardiac disease models have been developed to date using patient-specific iPSCs. These include LQTS (36), Brugada (37), CPVT (38), ARVC (39), dilated cardiomyopathy (40), hypertrophic cardiomyopathy (41), Andersen–Tawil syndrome (42), and Timothy syndrome (43) and have been recently reviewed (26). These initial studies were basic proof of principle exercises but have ushered in the capability of more complex disease investigation going forward. For example, genome editing technology with utilizing CRISPR/Cas9 system allows for the generation of isogenic lines (correction of the specific mutation site only) and allows for direct comparison excluding other genetic modifiers or epigenetic factors that may influence the cellular phenotype (44). Our group studied iPSC-CMs from a LQT2 patient with a hERG1 mutation H70R and compared it to both a control cell line and isogenic CRISPR- “corrected” iPSC-CMs (45). This methodology enabled us to identify a complex and unexpected cellular phenotype of hERG1a and hERG1B ratio imbalance in addition to mutant channel trafficking abnormality. These important physiologic nuances are not apparent in heterologous channel expression studies and highlight the strength of the iPSCs model.

Patient-specific genetic and transcriptional variation can manifest in iPSCs for more representative disease modeling, while non-human *in vivo* models (46–48) or other human cell models (i.e., HEK) unable to do so. Moreover, while most investigations of disease have been focused on studying iPSC-CMs, other cell types such as cardiac fibroblasts may also modulate disease features (19). Therefore, modeling the disease with patient-specific iPSC-CMs and iPSC-CFs that carry all the genetic variations and transcriptional regulation will provide us more comprehensive and in-depth understanding of human disease and the development of the model for drug screening and precision medicine.

Challenges of the iPSC-CMs investigation including the electrical, morphologic, and metabolic functionality are barriers to appropriate disease modeling. In the remainder of this commentary, we will summarize the progress made to overcome those challenges.

## Electrical maturity of IPSC-CMs

The rationale behind the use of iPSC-CMs is that they are human cells, patient-specific and can function as cardiomyocytes. Although advances have been made in the differentiation of iPSC-CMs, several features of electrical immaturity can manifest and limit their use for modeling cellular arrhythmia mechanisms and inherited arrhythmias.

**Figure 1:**
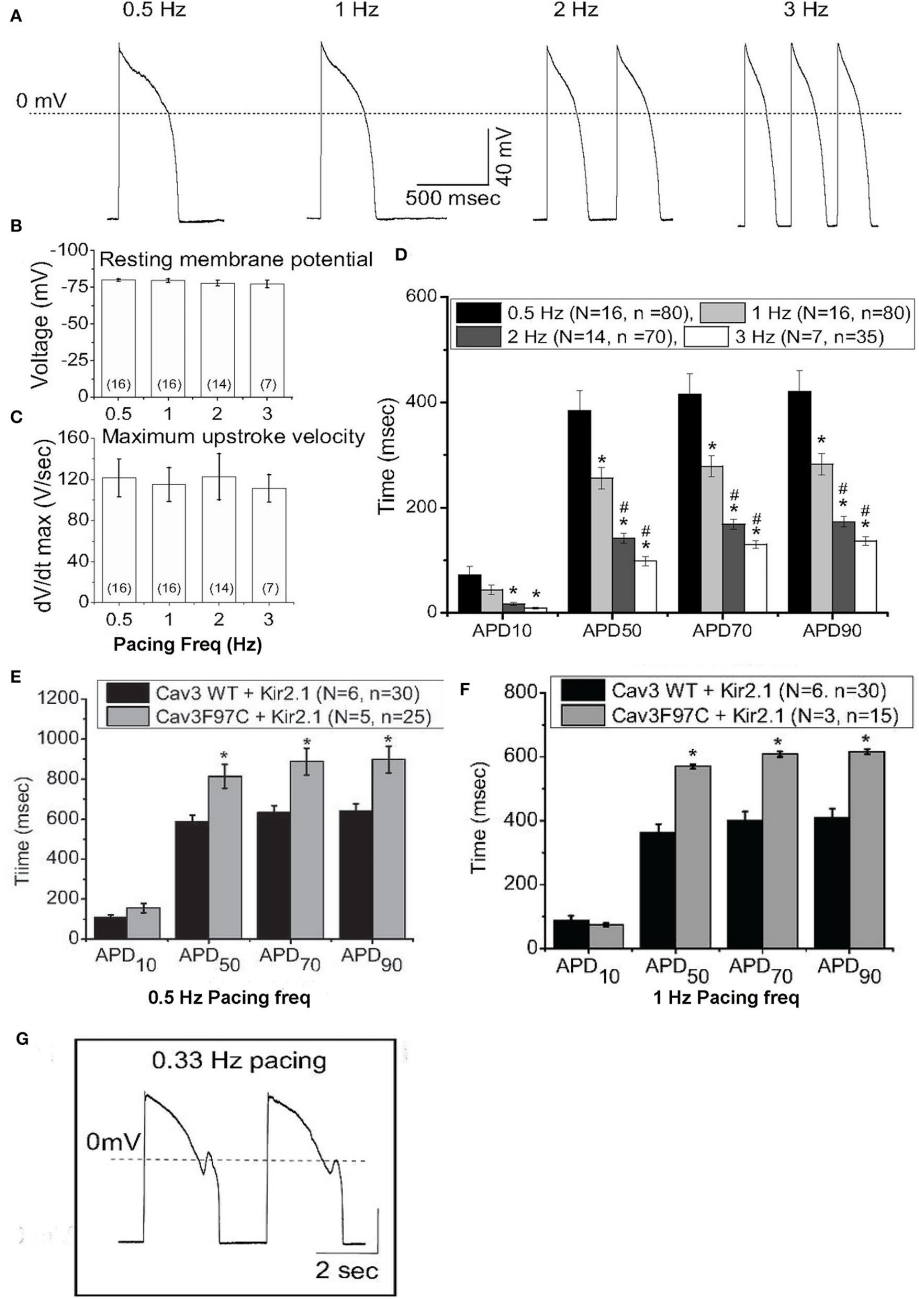
IK1 enhancement of iPS-CMs results in improved action potential (AP) characteristics. **(A)** Representative AP from IK1-enhanced iPS-CMs, paced at 0.5-3Hz at physiological temperature. **(B)** Resting membrane potential from IK1-enhanced iPS-CMs at different pacing frequencies. **(C)** dV/dTmax of iPS-CMs at different pacing frequencies. **(D)** Action potential duration (APD) at 10% (APD10), 50% (APD50), 70% (APD70) and 90% (APD90) at pacing frequencies 0.5Hz (black), 1Hz (dark grey), 2Hz (light grey), and 3Hz (white). **(E)** APD at APD10, APD50, APD70 and APD90 at 0.5Hz pacing from IK1 -enhanced iPS-CMs infected with WT Cav3 or LQT9-associated Cav3 mutation, F97C. **(F)** APD from IK1 -enhanced iPS-CMs expressing WT-Cav3 or F97C-Cav3 at 1Hz pacing. **(G)** Bradycardic pacing induced EADs in IK1-enhanced iPS-CMs expressing F97C-Cav3. Modified from (55).

Experimentally, iPSC-CMs exhibit a depolarized resting membrane potential and spontaneous automaticity due to small *I*_*K*1_and unchecked pacemaker *I*_*f*_ (49, 50). Kir2.1 is the dominant molecular component of *I*_*K*1_ that completes phase 3 repolarization and maintains resting membrane potential. In addition to electrophysiological functions, Kir2.1 is important for fetal mouse cardiomyocyte maturation (51) and important for facial muscular/skeletal development and growth (52). We and others have increased *I*_*K*1_ (Kir2.1 enhancement or current injection) in iPSC-CMs which results in control of cellular pacing and AP restitution, in agreement with most mammalian models (53–55). Additionally, increasing *I*_*K*1_ density establishes a more negative membrane potential, which increases the availability of sodium and L-type calcium channels, reflected in dV/d*t* values in the range of adult myocytes (55), as shown in Figures 1A–C, from Vaidyanathan et al. Additionally, action potential duration (APD) was more ventricular-like and exhibited rate adaptation in response to increased pacing frequency, typical of adult cardiomyocytes (Figure 1D). This also caused larger calcium transients compared to controls without changes in basal calcium levels or rate of decay of the transient. *I*_*K*1_-enhanced cells were not only more electrically mature but also induced maturation of other properties. Interestingly, unlike injection of current, enhancement of *I*_*K*1_ by expression of Kir2.1 resulted in an increase in capacitance and an increase in DNA synthesis, suggesting a larger role for channel expression beyond creating membrane polarization. Further, expression of the LQT9-associated mutation, Cav3-F97C, resulted in prolonged APD, and upon bradycardic pacing, EADs were observed (Figures 1E–G).

Several innovations have been developed to improve structural maturity and calcium handling (56). Prolonged culture time resulted in more mature phenotypes with respect to myofibril density, visible sarcomeres, calcium handling, and β-adrenergic response (57). Additionally, prolonged culture time allowed for upregulation of maturation-related genes. Increased gene expression of *I*_*K*1_ was reported; however, the increase is modest and remains several levels below what is observed in adult cardiac myocytes. Furthermore, the use of hormones, such as thyroid hormone and glucocorticoids, was reported to promote the development of t-tubules and larger calcium transients (12, 14). The combination of T3 and dexamethasone (a glucocorticoid analog) resulted in excitation–contraction coupling gain (12). Co-culture with human mesenchymal cells or treatment of iPSC-CMs with a collection of “paracrine factors” (including fibroblast growth factor, stromal cell-derived factor-1, and granulocyte–macrophage colony-stimulating factor) that in physiological conditions are secreted by neighboring cells shows some effects on markers of metabolism that resembles more mature cells (58, 59). Mechanical stress combined with pacing has been demonstrated to increase contractility, cell size, and RyR2 and SERCA2 expression (Ca^2+^ handling), pushing the cells to more mature excitation–contraction coupling (54, 55). Some exciting work has evolved in which the engineering of the culture substrate stiffness can yield more structurally mature iPSC-CMs (56, 57). The combination of both substrate stiffness and plating cell density functionally improves AP upstroke velocity, Kir2.1 expression, and markers of mature myofilaments (60). Harnessing the concept of neighboring cell cues growing in a syncytium has also been demonstrated to improve *I*_*K*1_ density (61). These important steps and technological advances continue to evolve and further iterations with a combination of methodologies are most likely needed to reach a truly electrical mature cell. Some of these will be discussed below in the section “Investigative Platforms.”

Testing of potential clinical pharmaceuticals for cardiac arrhythmogenesis is another use for iPSC-CMs. Drugs are routinely screened for their ability to produce Torsade de Pointes (TdP) in patients *via* preclinical and clinical trials and largely focus on blockade of hERG1 channel (62). Despite this screening, some drugs are excluded despite having no obvious ability to produce TdP and some are passed through but still can cause TdP. As a result, The Comprehensive *in vitro* Proarrhythmia Assay (CiPA) is being developed to allow an integrated, mechanistic risk assessment with strong evidence to inform regulatory decision-making and efficiency for drug discovery (62). Shortcomings of this approach abound, specifically since *I*_*K*1_ is largely absent from most iPSC-CM preparations and Kir2.1 is known to be blocked by numerous FDA-approved drugs (63–66). Further, TdP induction occurs with bradycardia/pause or with long–short cycles; thus, arrhythmia induction requires control of automaticity to provoke bradycardia or pace with long–short cycles (67). CiPA is an important step in the use of iPSC-CMs for drug safety; however, the lack of normal electrical physiology could overinflate some findings or miss key, potentially problematic effects on other ion channels. One workaround for this has been to inject *I*_*K*1_when assessing the AP characteristics (68). The utility of this approach is somewhat limited as the biological and regulatory aspects of Kir2.1 are missing from the model system. Another separate approach to this challenge is the emerging technology of human heart slices and recent developments that allow for mini-tissue-level drug toxicity studies. This multicellular approach poses some advantage for drug screening but is limited in scope by the availability of whole organs and limitations in culture and analytic outputs; for review, see Meki et al. (69).

## Cardiac metabolism in culture

Understanding iPSC-CM metabolism is key to generating a more mature cell for the study of human disease. One such approach is long-term culture of iPSC-CMs. In a pivotal study, Ebert et al. demonstrated that long-term culture (>200 days) results in divergent control of mitochondrial metabolism, regulated by PKA and proteasome-dependent signaling events (70). Additionally, heat shock protein 90 (Hsp90) worked downstream to regulate mitochondrial respiratory chain proteins and their metabolic output. This process increased iPSC-CM metabolism, resulting in increased cell contractility (70). Indeed, transitional states during development have provided insights into the role of mitochondria and metabolic transitions in iPSC-CMs. Future work demonstrating how long-term culture influences signaling cascades that control metabolic output and cellular homeostasis will further advance this work.

Developed mitochondria are central to cardiac metabolism, as they progress from small, fragmented mitochondria to large organelles that can produce enough ATP to sustain the contractile function of the heart (71). Mitochondria in immature iPSC-CMs are small and found throughout the cytoplasm with concentration in the perinuclear space, whereas mature mitochondria are larger and found between myofibrils and in the subsarcolemmal space (72). As the heart develops early during embryonic development, it can utilize a variety of substrates to meet its energy requirements as the embryo continues to develop (71). A key switch that is made during the perinatal transition is switching from glycolysis to oxidative phosphorylation to meet metabolic demands (73). Thus, developed mitochondria with active oxidative phosphorylation and the main energy driver for the cardiomyocyte are both a feature of metabolically mature iPSC-CMs and a key factor in initiating maturation (16, 74, 75).

The switch from glycolysis to mitochondrial oxidation in iPSC-CMs is vastly different from undifferentiated iPSCs, progenitors, and iPSC-derived non-CMs (74). Exploiting this difference is a major pathway to purifying iPSC-CMs following differentiation. Depriving glucose from differentiating iPSCs and iPSC-derived non-CMs leads to cell death, as they rely on glucose and glutamine metabolism. Using lactate supplementation, iPSC-CMs can survive without glucose and glutamine, thus allowing for their purification (76–78). However, the use of lactate was recently found to generate iPSC-CMs that have a similar phenotype to what is known to occur in ischemic heart failure (79). Therefore, simply replacing glucose with lactate in the culture media may not generate healthy iPSC-CMs during differentiation. Further exploiting metabolism to influence cell properties, glucose-deprived and fatty acid-supplemented culture media can facilitate the maturation of iPSC-CMs (80, 81). With the use of fatty acids, there is a risk for lipotoxicity, which can be avoided using galactose supplementation (82). The inclusion of glucose in the culture medium upregulates HIF1α-lactate dehydrogenase A axis and led to active glycolysis. Therefore, inhibition of HIF1α or lactate dehydrogenase A resulted in structural, metabolic, and electrophysiological maturation (82). It has also been reported that high-glucose culture conditions inhibit the maturation of iPSC-CMs *via* the pentose phosphate pathway (83).

Mitochondria also regulate micro-RNA (miRNA) levels that are involved in cardiomyocyte differentiation and maturation (84, 85). Time-dependent changes in miR-1 regulate electrophysiological maturity as iPSC-CMs mature (86). Therefore, it is possible that mitochondrial dynamics regulates this transition *in vivo*. It has been demonstrated that fluctuations in the redox state of a cell due to metabolism can impact membrane current in iPSC-CMs (87, 88). Additionally, downregulation of Opa1 resulted in increased mitochondrial fission, lower metabolic demand, and smaller, globular mitochondria in neonatal rat ventricular myocytes reprogrammed into pacemaker cells with TBX18 (89). Further, deletion of hydratase subunit A (HADHA) resulted in immature iPSC-CMs with fragmented mitochondria, accumulation of long-chain fatty acids, and prolonged action potential duration with no change in resting membrane potential (90). It appears that mitochondria can affect the electrical maturation of cardiomyocytes, but electrical stimulation of neonatal rat cardiomyocytes was demonstrated to regulate mitochondrial function by upregulating nuclear respiratory factor 1 (NRF-1), cytochrome oxidase, and carnitine palmitoyltransferase I (CPT-1) (91).

## Investigative platforms both 2D and 3D

Various techniques have been developed to culture iPSC-CMs in multidimensional constructs to achieve organizational and structure–function relationships that mimic native heart cells.

Utilizing advancements of the 2D platform is enticing as these are relatively cost-effective with reagents and techniques that are accessible. Such approaches can provide insight into cell function at molecular level, and 2D formats enable rapid perfusion with drugs for high-throughput screening and mechanical readouts or various imaging and optical mapping techniques. In traditional 2D formats, iPSC-CMs are grown as monolayers on glass or plastic substrates that have Young's modulus in the gigapascal (GPa) range. These conditions even with metabolic enhancements can result in less mature phenotypes in terms of structure, function, and gene expression response with limited ability to reflect *in vivo* dynamics of cardiac tissues (92). In contrast, iPSC-CMs cultured on physiologic soft substrate stiffness (93, 94) have improved contractile mechanics (95) with improved action potential and calcium-transient assessments (60). Using polydimethylsiloxane (PDMS) of the elasticity of normal adult myocardium can increase cell conduction velocity and contractile function.

**Figure 2:**
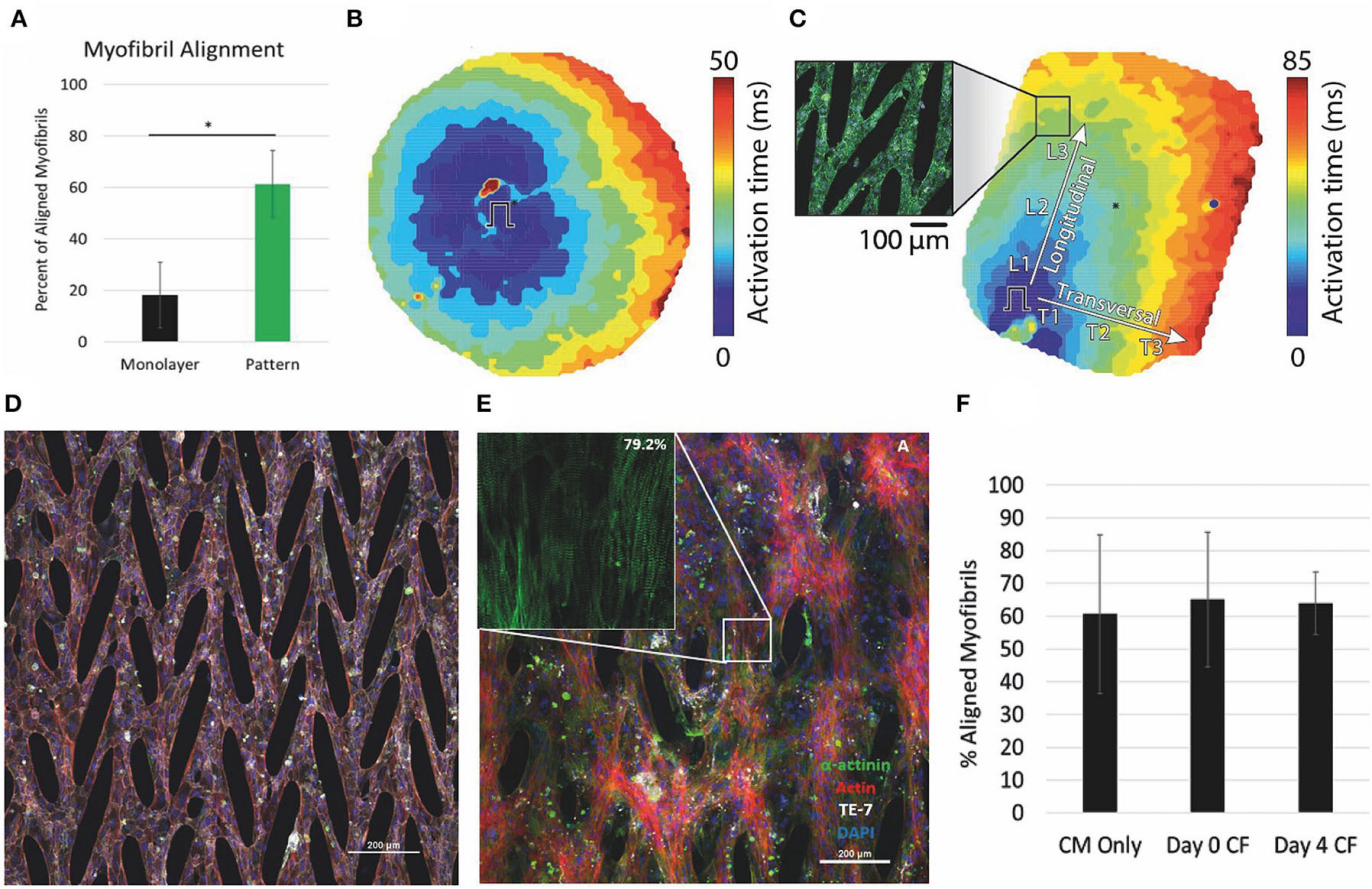
Micropattern culture of iPS-CMs results in anisotropic conduction and improved myofibril alignment, which is maintained in coculture with CFs. **(A)** Percent of myofibrils aligned within 10 degrees of the primary axis significantly different in monolayers vs micropatterned. **(B,C)** Optical mapping of **(B)** monolayer and **(C)** micropatterned iPS-CMs. Ansiotropic conduction in patterned determined by comparison of the longitudinal (CVL) and transverse (CVT) conduction velocities. **(D)** iPS-CMs cultured on micropattern alone. Scale bar 200μM. **(E)** Aligned myofibrils of iPS-CMs seeded on micropatterned substrate maintained in co-culture with CFs at 18 days. Inset shows myofibrils with 79% of sarcomeres within 10 degrees of the superior angle. Scale bar 200μM. **(F)** Quantification of the percent of myofibrils aligned within 10 degrees of the superior angle at 18 days for the three conditions: iPS-CMs cultured alone, iPS-CMs co-cultured with CFs from Day 0 seeding, iPS-CMs co-cultured with CFs from Day 4 of seeding. **(A–C)** modified from (97), **(D,E)** unpublished data, and **(F)** modified from (99).

Unique engineered substrates have also advanced 2D culture for iPSC-CMs. Micropatterns offer a mechanism to create a platform for cell connectivity and native sarcomere morphology (96). We and our collaborators have used micropatterning to create PDMS-stamped platforms for iPSC-CMs with highly aligned myofibrils (Figure 2A) (97). This powerful combination allowed for the formation of cellular syncytia with aligned myofibrils and demonstrated anisotropic conduction (Figures 2B,C) (97). Moreover, the recorded contractile strength was improved, and conduction velocities were 2x faster than in cells grown on PDMS as monolayers, likely due to the aligned cells and myofibrils in the patterned 2D monolayer. Disease models can also be created with patient-derived iPSC-CMs. In recent work, we show that the mechanical behavior of cardiomyocytes derived from a patient with CVPT and cultured on a micropatterned substrate is distinctly different from that of a familial control in both spontaneous rate of contraction and amplitude of contractile strain (98).

In the native heart, cardiomyocytes make up only part of the heterocellular composition. With the recent capability to create iPSC-cardiac fibroblasts (4), we have also coupled the 2D micropatterned platform with co-culture of iPSC-CMs and iPSC-CFs (Figures 2D–F) (99). The co-culture results in remodeling of the extracellular matrix (ECM) and improves calcium dynamics and contractile strain compared to iPSC-CMs on pattered substrate alone.

Beyond 2D culturing and planting platform advances, innovative 3D constructs have distinct advantages of cellular and structural heterogeneity to more closely mimic native cardiac tissue. Some examples include engineered heart tissue (EHT) cardioids, 3D bioprinting, biometric scaffolds such as biowires, bioreactors, and organ-on-a-chip microphysiological system (100, 101). Highlighting the strength of 3D systems to mimic cardiac contraction, several genetic disorders have been modeled using EHT including hypertrophic cardiomyopathy (102), left ventricular hypertrophy (103), dilated cardiomyopathy (104), and muscle dystrophy (105). Cardioids are sphere-shaped mini-organs that demonstrate self-organization, renewal, and differentiation abilities and are suitable to study early human cardiogenesis, injury regeneration (106), and congenital cardiac malformation (107). Though 3D platforms can yield interesting. results (13), drawbacks include the large number of required cells with lengthy and technical setup time. One alternative is the use of microtissues which are formed by mixing various cell types: cardiac fibroblast (CFs), endothelial cells (ECs), and cardiomyocytes (108). These more simplified constructs require considerably less start-up costs but lack more complex structural relationships between cells.

Going forward, the development of live-cell imaging techniques of intercellular Ca^2+^, sarcomere lengths, and membrane potential will help the functionality of 3D platform (109). Furthermore, development of methods allowing exposure of all cell layers to therapeutics or toxins will be instrumental to increasing the functionality of 3D platforms.

## Conclusion

Over the last two decades, substantial progress has been made in generation, culture, and application of iPSC-CMs. Using iPSC-CM model to recapitulate the complex genetic background of disease entities provides an indispensable platform to study protein–protein and structure–function relationships. Furthermore, the use of human cells and the development of techniques to model the physiological environment may even someday eliminate the need for animal models. As we push the boundaries of iPSC-CM technology, we come closer to a better understanding of human cardiac disease toward refined approaches to therapeutics.

## Author contributions

LR contributed to manuscript writing, figure production, and editing. SM contributed to writing, figure production, and editing. JZ contributed to manuscript writing and editing. WC and LE contributed to project conceptualization, writing, figure production, and editing. All authors contributed to the article and approved the submitted version.

## Funding

This project was funded in part by (1R01HL163987-01, 1R01HL139738-01A1, and 1R01HL128598-01 to LE), Karen Thompson Medhi Professorship to WC, Office of the Vice Chancellor for Research and Graduate Education to WC, and the Gary and Marie Weiner Professor in Cardiovascular Medicine Research to LE.

## Conflict of interest

The authors declare that the research was conducted in the absence of any commercial or financial relationships that could be construed as a potential conflict of interest.

## Publisher's note

All claims expressed in this article are solely those of the authors and do not necessarily represent those of their affiliated organizations, or those of the publisher, the editors and the reviewers. Any product that may be evaluated in this article, or claim that may be made by its manufacturer, is not guaranteed or endorsed by the publisher.
